# Editorial: Diagnosis, monitoring, and treatment of heart rhythm: new insights and novel computational methods

**DOI:** 10.3389/fphys.2023.1272377

**Published:** 2023-08-16

**Authors:** Jieyun Bai, Jichao Zhao, Haibo Ni, Dechun Yin

**Affiliations:** ^1^ Department of Electronic Engineering, College of Information Science and Technology, Jinan University, Guangzhou, China; ^2^ Auckland Bioengineering Institute, University of Auckland, Auckland, New Zealand; ^3^ Department of Pharmacology, University of California Davis, Davis, CA, United States; ^4^ Department of Cardiology, The First Affiliated Hospital of Harbin Medical University, Harbin, China

**Keywords:** cardiac arrhythmias, artificial intelligence, computational modelling, deep learning, digital twin, ECG

## 1 Introduction

The human heart, a marvel of precision and complexity, is governed by rhythmic electrical impulses that orchestrate its regular contractions, propelling life-sustaining blood throughout the body. However, disruptions to this intricate electrical system can lead to arrhythmias (i.e., heart rhythm disorders), which can have serious consequences for an individual’s health and wellbeing. Heart rhythm disorders encompass a wide range of conditions that affect the heart’s electrical system, leading to irregular heartbeats, either too fast (tachycardia) or too slow (bradycardia), or chaotic rhythms. These disorders can cause symptoms and predispose to conditions ranging from palpitations, dizziness, and shortness of breath to more severe consequences, such as heart failure, stroke, or sudden cardiac death (Conti, 2019). As a leading cause of morbidity and mortality worldwide, heart rhythm disorders pose a substantial burden on healthcare systems and society at large (Nabel, 2003).

Arrhythmias remain a complex and challenging area in medicine. While significant progress has been made in understanding and treating arrhythmias, there are several reasons that contribute to the ongoing challenges in diagnosing and treating these conditions effectively (Offerhaus et al., 2020): 1) The cardiac electrical system is intricate, and arrhythmias can arise from various mechanisms, making their diagnosis and treatment challenging (Zeppenfeld et al., 2022); 2) Despite advancements in cardiac electrophysiology, substantial gaps still exist in our understanding of the precise mechanisms that lead to certain arrhythmias (Dobrev et al., 2019); 3) Arrhythmias can present in different forms and affect different regions of the heart, making it challenging to develop a one-size-fits-all treatment approach; 4) Some forms of arrhythmias may be intermittent and thus difficult to capture during routine clinical evaluation, leading to underdiagnosis or delayed diagnosis (Kirchhof, 2017); 5) Each patient’s response to treatment can vary substantially due to differences in their physiology, genetics, and co-existing medical conditions (Ni et al., 2018); 6) Anti-arrhythmic medications may even cause or worsen arrhythmias, requiring a carefully balanced evaluation between risk and benefit (Vicente et al., 2018); 7) While invasive procedures like catheter ablation can be effective, they come with inherent risks and may not be suitable for all patients (Ramanathan et al., 2004); 8) The current treatment strategies often rely on a trial-and-error approach, and there is a need for more personalized and targeted therapies; 9) Some arrhythmias are caused by scar tissue in the heart, which can be challenging to treat and manage effectively; and 10) Detecting and diagnosing arrhythmias can be challenging if patients are not aware of their symptoms or do not seek medical attention promptly. Therefore, this Research Topic collected a series of reviews and original research articles presenting recent advances toward a better understanding, diagnosis and treatment of cardiac arrhythmias, including: 1) structure-detailed computer modelling; 2) biophysics-based computer modelling; 3) biosignal-based diagnostic and monitoring; and 4) population-based statistics and new therapeutic frameworks. A total of 28 accepted articles were published under this Research Topic. Here in this editorial, we summarize the new knowledge and approaches generated, and discuss how these can contribute to an improved understanding of heart rhythm and clinical treatment, as well as how they may provide insights into future research directions.

## 2 Structure-detailed computer modelling

The heart’s complex structure and function play a central role in maintaining blood circulation, making it crucial to understand its mechanisms and potential dysfunctions (Hansen et al., 2015). Over the years, advancements in technology have paved the way for sophisticated computer modelling techniques, enabling researchers to create detailed simulations of the heart’s structure and function. Structure-detailed computer modelling allows for the creation of highly accurate representations of the heart’s anatomy. By combining medical imaging data, such as magnetic resonance imaging (MRI) and computed tomography scans, with computational techniques, researchers can build three-dimensional models that precisely mimic the heart’s architecture (Bai et al., 2023). These models provide a valuable tool for visualizing and analyzing the intricate organization of cardiac tissues (Zhao et al., 2012), including the ventricles, atria, valves, and the conduction system (Xiong et al., 2021). In order to accurately obtain the anatomical structure of human heart from MRI, researchers proposed deep learning models. For example, Xiong et al. proposed a novel deep learning framework for 3D surface reconstruction of the left atrium directly from point clouds acquired through clinical mapping systems during cardiac ablation. In contrast, Chen et al. focused on accurate segmentation of the ventricle and myocardium in cardiac MRI proposed a dilated convolution network with an edge fusion block and directional feature maps. This is a critical step in evaluating cardiac function. It is important to note that while artificial intelligence (AI) holds great promise in cardiac imaging, its integration into clinical practice requires rigorous validation and regulatory approval to ensure safety and efficacy. Collaborative efforts between AI developers, medical professionals, and regulatory bodies are crucial to harness the full potential of AI in cardiac imaging and other medical domains.

## 3 Biophysics-based computer modelling

Biophysics-based modelling of the heart represents an innovative approach that combines principles from physics, mathematics, and biology to create comprehensive simulations of the heart’s behavior (Clayton et al., 2020). By leveraging biophysical data and computational techniques, researchers can gain a deeper understanding of the heart’s intricate dynamics at various scales. Many researchers conducted multi-scale cardiac electrophysiology modeling, providing valuable insights into the underlying physiological processes and helping guide the development of new therapeutic approaches for cardiac arrhythmias and other related conditions (Colman et al., 2017; Ni et al., 2020; Morotti et al., 2021). For example, Jin et al. used computational modeling to explore the effects of ablation and antiarrhythmic drugs on patients with PITX2 gene deficiencies in atrial fibrillation (AF) (Bai et al., 2019; 2020b; Bai et al., 2021a; Zhu et al., 2021). Virtual simulations demonstrate that certain antiarrhythmic drugs have more significant effects in patients with PITX2 deficiencies (Bai et al., 2021b), providing insights into tailored treatment strategies (Bai et al., 2020a). Jiang et al. also evaluated the efficacy of common antiarrhythmic drugs and specific *I*_*Kr*_ activators for treating arrhythmias induced by carbon monoxide (CO) in healthy and failing hearts. Simulation results indicate that the tested antiarrhythmic drugs are not effective against CO-induced arrhythmias, whereas *I*_*Kr*_ activators show promise for treatment. In a study by Li et al., a mathematical model was developed to simulate the effect of arsenic trioxide (ATO) on ventricular electrical excitation at cellular and tissue levels. The study revealed how ATO-induced alterations in ion channels lead to prolonged action potential duration and increased risk of arrhythmias, providing insights into potential pharmacological intervention.

Some studies aimed to develop powerful tools for researchers and clinicians to gain insights into the complex electrical behavior of the heart from cellular level to the organ level. For instance, Yang et al. proposed a WebGL-based framework to visualize the three-dimensional synergetic biological modality of the heart, combining physical volume data and electrophysiological modality. Galappaththige et al. developed a computational modeling framework to rigorously evaluate the performance of cardiac mapping systems. The framework provides a quantitative analysis of mapping system performance, aiding in system accuracy estimation. In personalized medicine, Bai et al. discussed the role of digital twin techniques, combining mechanistic and statistical models, in advancing research on atrial fibrillation. It highlighted their applications in understanding AF mechanisms, screening anti-AF drugs, and optimizing ablation strategies, emphasizing the potential transition from AF description to response prediction. Aside from focusing on the electrical properties of the heart, Sanatkhani et al. used computational fluid dynamics to examine the effects of subject-specific factors on the residence time distribution of blood particles in the left atrial appendage in atrial fibrillation. These modeling studies showcase the power of computational techniques in improving our understanding of cardiac arrhythmias and their treatment, potentially paving the way for more personalized and effective therapeutic approaches in the future.

## 4 Biosignal-based diagnostic and monitoring

With the advent of advanced signal processing techniques, researchers have been able to extract valuable insights from electrocardiogram (ECG) data and other cardiac signals. Several studies of this Research Topic developed AI-powered algorithms for detecting arrhythmias using ECG data. For example, Wu et al. presented an automatic system combining denoising and segmentation modules to detect ST-segment and J-point deviation from Holter ECG data. The ECG Bidirectional Transformer network was used for denoising and segmentation tasks, achieving high precision in detecting subtle ST-segment changes in noisy ECG signals. Different from the feature extraction of ECG, Huang et al. developed diagnostic models to identify individuals with AF using amplified sinus-P-wave analysis. Zhang et al. designed a screening algorithm to distinguish different types of premature beats from paroxysmal AF in ECG segments. The proposed method effectively eliminates single and other types of premature beats to improve the accuracy of paroxysmal AF detection. The algorithm was validated on different databases and achieved high accuracy, providing potential for real-time analysis using wearable devices. Based on ECG data, different perdition models based on deep learning also were proposed (Zhang et al.; Zhang et al.). Recently, internet of things (IoT)-based ECG monitoring shows a great potential for patient-centric, connected, and data-driven cardiac care. However, signal quality is a critical factor that can significantly impact the overall performance and functionality of the IoT system. Therefore, Liu et al. introduce a new method for assessing the quality of wearable ECG signals using wavelet scattering and long short-term memory network. Different from ECG signals, signals of arterial blood pressure Chou et al., pulsed ultrasound (Xiao et al.; Deng et al.), skin sympathetic nerve activity Cai et al., impulse radio ultra-wideband radar Qiao et al. and photoplethysmogram Sološenko et al. were also used to improve the understanding the function of the heart and diagnosing abnormal heart rhythms. These new monitoring and diagnostic methods continued to advance, offering more efficient and powerful techniques to extract valuable information from cardiac signals.

## 5 Meta-analysis and clinical studies

Meta-analysis and clinical studies in heart rhythm have been pivotal in advancing our understanding of cardiac arrhythmias (Wang et al., 2021) and guiding evidence-based clinical decision-making. Several novel key findings on arrhythmias were noted in this Research Topic. Hashimoto et al. investigated the incidence of arrhythmias in healthy volunteers of varying ages using ambulatory electrocardiography. Their study revealed that ventricular and supraventricular ectopy increased with age, and aging significantly influenced the frequency of ventricular ectopy. Additionally, age, body mass index, and heart rate variability were associated with supraventricular ectopy, providing age-specific reference intervals for ectopy in healthy individuals. Wei et al. conducted a retrospective analysis on post-cryoballoon ablation (CBA) patients and developed a machine learning-based nomogram to predict the risk of atrial fibrillation (AF) recurrence. Their predictive model outperformed conventional risk scores, offering a valuable tool for personalized treatment decisions and improved patient outcomes. A meta-analysis by Liu et al. evaluated the effect of sacubitril/valsartan therapy on cardiac arrhythmias and the risk of sudden cardiac death in heart failure patients. The analysis demonstrated a promising reduction in the risk of sudden cardiac death compared to the control group, suggesting potential anti-arrhythmic properties of sacubitril/valsartan in heart failure management. Li et al. developed a nomogram to predict the risk of new-onset atrial fibrillation in septic patients. The model, which incorporated various clinical risk factors, demonstrated excellent predictive accuracy, particularly in septic shock patients, aiding early risk assessment and individualized treatment strategies. Han et al. developed the HASBLP score, a predictive model to identify AF patients at higher risk of recurrence after catheter ablation. The score outperformed existing risk scores and provides clinicians with a valuable tool for predicting AF recurrence and guiding personalized follow-up and treatment plans. Liu et al. investigated the role of I-κB kinase-ε (IKKε) in doxorubicin-induced dilated cardiomyopathy (DCM). Their experiments showed that IKKε deficiency improved cardiac function, suggesting IKKε as a potential therapeutic target for managing this condition. Finally, Wang et al. provided a systematic review of scTdP (short-coupled variant of torsade de pointes), exploring its clinical features, diagnosis, and management. Further large-scale studies are needed to clarify existing arrhythmogenic entities. Overall, these studies collectively advance our knowledge of heart rhythm disorders, enhancing patient outcomes, and guiding clinical guidelines and practice in the field of cardiology.

## 6 Conclusions and future directions

The research presented in this Research Topic has contributed significantly to our understanding of cardiac arrhythmias and has shed new lights on potential improvements in diagnosis and treatment. Continued efforts in multidisciplinary research, technology integration, and personalized approaches hold the potential to revolutionize arrhythmia care and improve the quality of life for millions of patients worldwide. Through collaboration and continued exploration, we can look forward to a future where arrhythmia diagnosis and treatment are more accurate, effective, and accessible to all individuals in need.
